# Lipid disorders among Black Africans non-users of lipid-lowering medication

**DOI:** 10.20945/2359-3997000000076

**Published:** 2018-10-01

**Authors:** Mariana Veronez Borgo, Marcelo Perim Baldo, Rafael de Oliveira Alvim, Divanei Zaniqueli, Daniel Pires Capingana, Pedro Magalhães, Amilcar Bernardo da Silva, Polyana Romano Oliosa, Carmem Luiza Sartório, José Geraldo Mill

**Affiliations:** 1 Universidade Federal do Espírito Santo Universidade Federal do Espírito Santo Programa de Pós-Graduação em Saúde Coletiva Vitória ES Brasil Programa de Pós-Graduação em Saúde Coletiva, Universidade Federal do Espírito Santo (UFES), Vitória, ES, Brasil; 2 Universidade Estadual de Montes Claros Universidade Estadual de Montes Claros Departamento de Fisiopatologia Montes Claros MG Brasil Departamento de Fisiopatologia, Universidade Estadual de Montes Claros (Unimontes), Montes Claros, MG, Brasil; 3 Universidade Federal do Espírito Santo Universidade Federal do Espírito Santo Programa de Pós-Graduação em Ciências Fisiológicas Vitória ES Brasil Programa de Pós-Graduação em Ciências Fisiológicas, Universidade Federal do Espírito Santo (UFES), Vitória, ES, Brasil; 4 Universidade Federal do Espírito Santo Universidade Federal do Espírito Santo Programa de Pós-Graduação em Nutrição e Saúde Vitória ES Brasil Programa de Pós-Graduação em Nutrição e Saúde, Universidade Federal do Espírito Santo (UFES), Vitória, ES, Brasil; 5 Universidade Agostinho Neto Universidade Agostinho Neto Escola de Medicina Departamento de Ciências Fisiológicas Luanda Angola Departamento de Ciências Fisiológicas, Escola de Medicina, Universidade Agostinho Neto (UAN), Luanda, Angola

**Keywords:** Lipid disorders, Black Africans, lipid-lowering medication

## Abstract

**Objective:**

Angola is a sub-Saharan African country where the population has scarce access to lipidlowering medication. We sought to determine the frequency of lipid disorders among Angolan nonusers of lipid-lowering medication.

**Material and methods:**

A cross-sectional descriptive study was carried out in a sample of 604 workers from the public sector. Blood pressure and anthropometric data were measured along with biochemical parameters including total cholesterol (TC), triglycerides (TG), low-density lipoprotein cholesterol (LDL-C) and high-density lipoprotein cholesterol (HDL-C). LDL-C to HDL-C ratio (LDL-C/HDL-C) was obtained from LDL-C and HDL-C levels.

**Results:**

High frequencies of elevated blood pressure (44.8%), metabolic syndrome (20.2%), increased TC (39.2%) and increased LDL-C (19.3%) were found. Low HDL-C was more frequent in women (62.4% *vs.* 36.1%, *p* < 0.001). Isolated hypercholesterolemia was more frequent in men (9.6% *vs.* 2.5%, *p* < 0.001). Among men TC, TG, LDL-C and LDL-C/HDL-C ratio were higher and HDL-C was lower in obese than in low-weight and normal-weight participants. Among women TC, TG, LDL-C and LDL-C/HDL-C ratio were higher in obese than in normal-weight participants. Significant linear trend of increasing TC and LDL-C levels as age increased was detected for both genders (*p* for trend < 0.05).

**Conclusion:**

The results of our study showed a high frequency of lipid disorders in Angolan non-users of lipid-lowering medication.

## INTRODUCTION

Dyslipidemia is a multifactorial lipid disorder that is dependent on genetic, environmental and lifestyle factors and is described as one of the most relevant risk factors for cardiovascular diseases (1). Indeed, dyslipidemia has been identified as one of the major modifiable risk factors for ischemic heart disease among young adults (2).

The definition of dyslipidemia is the presence of abnormal blood lipid levels, encompassing increased plasma levels of total cholesterol (TC), low-density lipoprotein (LDL-C) and triglycerides (TG), as well as decreased levels of high-density lipoprotein (HDL-C) (3).

In sub-Saharan Africa, the majority of countries is experiencing a rapid demographic and epidemiological : transition (4,5). Several studies have reported the prevalence of cardiovascular risk factors in sub-Saharan African populations (6–8), but these data are limited to a few countries (9,10).

Angola is a sub-Saharan African country where in the last few years, significant economic growth and increased urbanization has occurred (11). These changes may imply an increasing prevalence of diverse cardiovascular risk factors such as obesity, insufficient physical activity, high blood pressure and dyslipidemia.

The Angolan population has scarce access to lipidlowering pharmacological therapy, which offers an ideal opportunity to analyze the lipid profile in a large sample with minimal interfering factors. Therefore, in this study, we sought to determine the frequency of lipid disorders in an Angolan population consisting of non-users of lipid-lowering medication.

## MATERIAL AND METHODS

### Study design

This cross-sectional descriptive study was carried out in a sample consisting of civil servants working at Agostinho Neto University (UAN) in Luanda, Angola. The survey was conducted in the Department of Physiology from the Faculty of Medicine. All data were collected from 2009 to 2010, and details of the study design were described elsewhere (12,13). The project was approved by the Independent Ethics Committee on Research of the Faculty of Medicine of Agostinho Neto University following the standard procedures in human research in accordance with the Declaration of Helsinki.

Subjects ≥ 20 years of age working at UAN were invited to participate in a survey of cardiovascular risk factors. From the eligible sample comprising 1,458 staff members, 614 (42%) responded to the invitation. Only nine subjects (1.4%) were excluded from the present analysis due to use of lipid-lowering medication.

Demographics including socioeconomic class, educational level and medical history were collected during an interview using a structured questionnaire, as previously reported (14). Participants were classified as non-smokers (never and former smokers) and current smokers (daily and occasional smokers).

Socioeconomic classes were categorized into quartiles according to average monthly household income: first quartile (low class), second quartile (middle class), third quartile (upper middle class) and fourth quartile (upper class). Education levels were classified into three categories based on the number of years of education: low (≤ four years of education), middle (five to 12 years of education) and high (≥ 13 years of education) (13).

### Biochemical analyses

Participants reported to the Faculty of Medicine after 12h of fasting. They were asked to refrain from smoking, physical exercise and caffeinated beverages for at least 12 hours before the visit. Clinical exams were performed in a temperature-controlled room (22°C – 23°C) between 8 a.m. and noon. For determination of serum levels of TC, TG, HDL-C and glucose, venous blood was obtained by standard *forearm venipuncture* and processed immediately using commercially available kits (Biosystems S.A. Costa Brava 30, Barcelona, Spain).

All biochemical parameters were analyzed by enzymatic methods with a spectrophotometer (Biosystems BTS 310, Barcelona, Spain). LDL-C was calculated as previously described (15), and very low-density lipoprotein cholesterol (VLDL-C) was calculated as triglycerides/5 for those participants with TG < 400 mg/dL according to the Third Report of the National Cholesterol Education Program Expert Panel on detection, evaluation and treatment of high blood cholesterol in adults (NCEP-ATP III) (16). The LDL-C to HDL-C ratio (LDL-C/HDL-C) was obtained from LDL-C and HDL-C levels.

Diabetes mellitus was defined as fasting glucose ≥ 126 mg/dL or the use of antidiabetic drugs (17).

Dyslipidemia was classified into four phenotypes in accordance with V Brazilian Guidelines on Dyslipidemia and Atherosclerosis: isolated hypertriglyceridemia (TG ≥ 150 mg/dL), isolated hypercholesterolemia (LDL ≥ 160 mg/dL), mixed hyperlipidemia (TG > 150 and TC ≥ 200 mg/dL) and low HDL-C (isolated reduction of HDL-C, men < 40 mg/dL and women < 50 mg/dL or combined with either increased LDL-C or increased TG) (18).

Metabolic syndrome was defined based on the presence of three or more of the following conditions: waist circumference (WC) > 102 cm (men) or > 88 cm (women), systolic blood pressure (SBP) ≥ 130 mmHg and/or diastolic blood pressure (DBP) ≥ 85 mmHg and/or BP-lowering treatment, fasting triglyceride levels ≥ 150 mg/dL (1.70 mmol/l) or treatment for hypertriglyceridemia, HDL-C < 40 mg/dL (1.04 mmol/l) (men) or < 50 mg/dL (1.30 mmol/l) (women), treatment for dyslipidemia, fasting glucose level ≥ 110 mg/dL or use of anti-diabetic medication (16).

### Anthropometric measurements

Anthropometric measures of weight, height, WC and hip circumference (HC) were obtained with participants barefoot and wearing only underwear. Weight was measured to the nearest 0.1 kg using a previously calibrated mechanical scale (SECA GmbH & Co, Germany). A stadiometer fixed to the scale was used to measure body height to the nearest 0.5 cm.

WC and HC were measured twice using an inextensible 1-cm-wide tape measure. WC was measured at the end of a normal expiration at the midpoint between the lower border of the rib cage and the top of the iliac crest (19). The waist-to-hip ratio (WHR) was calculated from the WC and HC.

BMI was calculated as the weight in kilograms divided by height in meters squared (kg/m^2^). According to the BMI values, the participants were classified as low weight (< 18.5 kg/m^2^), normal weight (18.5-24.9 kg/m^2^), overweight (25.0-29.9 kg/m^2^) and obese (≥ 30.0 kg/m^2^) (20).

### Hemodynamic measurements

Blood pressure measurement was performed after a five-minute rest in triplicate in the non-dominant arm with the arm at the level of the heart. Systolic blood pressure (SBP), diastolic blood pressure (DBP) and HR were obtained using a validated, automated digital oscillometric sphygmomanometer (Omron 705CP, Tokyo, Japan). The readings were repeated at three- minute intervals. The mean of the last two readings was recorded. Elevated blood pressure was defined as SBP ≥ 140 mmHg and/or DBP ≥ 90 mmHg and/or the use of antihypertensive drugs.

### Statistical analysis

Statistical analysis was performed using SPSS software, version 20.0 (SPSS Inc., Chicago, IL, USA). The data's normality was examined using the Kolmogorov- Smirnov test. Continuous variables were reported as mean ± standard deviation (SD) or as proportions.

Student's *t* test was used to compare the means of two groups. Linear association between lipid fractions and categories of nutritional status was tested with ANCOVA. The chi-squared test was used to compare proportions. A test for linear trends (Jonckheere- Terpstra test) was performed to test the linear association between the age range as the independent variable and lipid fractions as the dependent variable. Normal distribution curves of total cholesterol and triglycerides were drawn separately for gender and median (P^50th^) with corresponding interquartile intervals (P^25th^ – P^75th^) provided. The level of significance was set at *p* < 0.05.

## RESULTS

Demographic data are summarized in Table 1. As expected, the sample comprised predominantly black individuals. Regarding educational level and socioeconomic status, approximately 60% had at least five years of formal education, and almost 68% were classified as members of the middle/upper socioeconomic class.

**Table 1:** Demographic characteristics of sample

		N	%
Gender			
	Male	291	48
	Female	314	52
	Total	605	100
Age range (years)		
	22-31	88	14.5
	32-41	137	22.6
	42-51	207	34.2
	52-61	149	24.6
	62-72	24	4.0
Race/ethnicity		
	White	5	0.8
	Black	577	95.4
	Mulattos	23	3.8
Education level		
	Low	243	40.2
	Medium	150	24.8
	High	212	35.0
Socioeconomic Class		
	Low	191	31.6
	Middle	203	33.6
	Upper	209	34.5
	MS	2	0.3

MS: missing data.

Table 2 shows the sample's general characteristics, emphasizing the comparison between genders. The age was similar between genders, but higher BMI and WC were observed in women than in men. A significant gender difference was not detected for SBP and DBP. Moreover, lipid profile was similar between genders, but HDL-C was higher in women than in men (*p* < 0.001).

**Table 2:** Anthropometric and biochemical characteristics of sample stratified by gender

	Male	Female	*p value*
Age (years)	45.1 ± 11.1	43.8 ± 10.0	0.18
Weight (kg)	67.7 ± 4.7	69.1 ± 15.7	0.33
Height (cm)	167 ± 7.0	159.5 ± 6.6	< 0.001
BMI (kg/m^2^)	24.0 ± 4.2	27.1 ± 5.8	< 0.001
WC (cm)	79.9 ± 12.8	83.7 ± 13.5	< 0.001
HC (cm)	91.4 ± 9.4	99.5 ± 1.5	< 0.001
WHR	0.87 ± 0.08	0.84 ± 0.09	< 0.001
SBP (mmHg)	137 ± 23	133 ± 27	0.09
DBP (mmHg)	83 ± 14	82 ± 14	0.86
Heart rate (bpm)	67 ± 10	69 ± 10	0.003
Glucose (mg/dL)	95 ± 20	92 ± 19	0.31
TC (mg/dL)	190 ± 42	192 ± 36	0.24
LDL-C (mg/dL)	125 ± 41	125 ± 38	0.79
HDL-C (mg/dL)	44 ± 10	48 ± 11	< 0.001
TG (mg/dL)	101 ± 42	99 ± 38	0.34
VLDL-C (mg/dL)	20± 8	20 ± 7	0.34

BMI: body mass index; WC: waist circumference; HC: hip circumference; WHR: waist-to hip ratio; SBP: systolic blood pressure; DBP: diastolic blood pressure; TC: total cholesterol; LDL-C: low-density lipoprotein cholesterol; HDL-C: high-density lipoprotein cholesterol; VLDL-C: very low-density lipoprotein cholesterol; TG: triglycerides.

Overall, a high frequency of elevated blood pressure (44.8%), metabolic syndrome (20.2%), increased TC (39.2%) and increased LDL-C (19.3%) was found. Moreover, gender interfered with the frequency of some risk factors. Obesity, metabolic syndrome and low HDL-C were more frequent in women than in men. On the other hand, smoking and isolated hypercholesterolemia were more frequent in men than in women (Table 3).

**Table 3:** Risk factors in the sample and stratified by gender

	All	Male	Female	*p value*
Elevated blood pressure	44.8	46.0	43.6	0.30
Diabetes	5.1	5.2	5.1	0.56
Obesity	19.2	8.9	28.7	< 0.001
Metabolic Syndrome	20.2	16.2	23.9	0.01
Smoking	6.1	8.2	4.1	0.03
TC ≥ 200	39.2	38.8	39.5	0.47
Isolated hypercholesterolemia	6.0	9.6	2.5	< 0.001
LDL-C ≥ 160 mg/dL	19.3	20.6	18.2	0.25
Isolated hypertriglyceridemia	4.6	6.2	3.2	0.06
TG ≥ 150 mg/dL	10.7	12.4	9.2	0.13
Mixed hyperlipidemia	1.7	1.7	1.6	0.903
Low-HDL-C	49.8	36.1	62.4	< 0.001

Data are presented as proportions (%). Elevated blood pressure: SBP ≥ 140 mmHg and/or DBP ≥ 90 mmHg or normotensive on antihypertensive medication. Diabetes: fasting glucose ≥ 126 mg/dL or on medication. TC, total cholesterol; LDL-c, low-density lipoprotein cholesterol; HDL-c, high density lipoprotein cholesterol; TG, triglycerides. Isolated hypercholesterolemia: isolated elevation of LDL-C (≥ 160 mg/dL); Isolated hypertriglyceridemia: isolated elevation of TGs (≥ 150 mg/dL); Mixed hyperlipidemia: Increased LDL-C (≥ 160 mg/dL) combined with increased TG (≥ 150 mg/dL). Low HDL-C: isolated reduction of HDL-C (Men < 40 mg/dL, Women < 50 mg/dL) or in association with elevated LDL-C and TG.

Table 4 shows the association between lipid fraction and nutritional status for both genders. Among men, TC, TG, LDL-C and the LDL-C/HDL-C ratio were higher, and HDL-C was lower in obese participants than in low-weight and normal-weight participants. A similar difference was found in overweight participants compared with low-weight participants, but in TG levels, such a difference was not detected between overweight and normal-weight men. On the other hand, among women, TC and LDL-C were higher in obese and overweight participants than in normal-weight participants, and TG was higher in obese participants than in low-weight and normal- weight participants. The LDL-C/HDL-C ratio was higher in obese participants than in normal-weight participants, but HDL-C was not different across the categories of nutritional status.

**Table 4:** Association between lipid fraction and weight status

	Low weight	Normal weight	Overweight	Obese
**Male**				
n (%)	16 (5.6)	168 (58.3)	78 (27.1)	26 (9.0)
TC (mg/dL)	168 ± 26	184 ± 39	199 ± 46*+	214 ± 33*+
TG (mg/dL)	78 ± 34	98 ± 36	108 ± 49+	123 ± 46*+
LDL-C (mg/dL)	104 ± 25	119 ± 39	135 ± 46*+	149 ± 36*+
HDL-C (mg/dL)	48 ± 14	46 ± 10	42 ± 10*+	40 ± 8*^+^
LDL-C/HDL-C	2.4 ± 1.0	2.8 ± 1.3	3.4 ± 1.4*+	3.9 ± 1.5*+
**Female**				
n (%)	11(3.6)	111(36.4)	93 (30.5)	90 (29.5)
TC (mg/dL)	191± 29	186 ± 35	196 ± 33*	197 ± 39*
TG (mg/dL)	88 ± 37	94 ± 34	97 ± 40	109 ± 41**
LDL-C (mg/dL)	125 ± 29	118 ± 35	129 ± 34*	129 ± 44*
HDL-C (mg/dL)	48 ± 7	49 ± 11	47 ± 12	46 ± 11
LDL-C/HDL-C	2.7 ± 0.7	2.6 ± 1.1	2.9 ± 1.2	3.1 ± 1.5*

Data are mean ± standard deviation. TC: total cholesterol; TG: triglycerides; LDL-C: low density lipoprotein cholesterol; HDL-C: high density lipoprotein cholesterol; LDL-C/HDL-C: LDL-C to HDL-C ratio. *p < 0.05 *vs.* normal weight, *p < 0.05 vs. overweight,^†^*p* < 0.05 vs. low weight. Comparisons adjusted for age.

**Figure 1:**
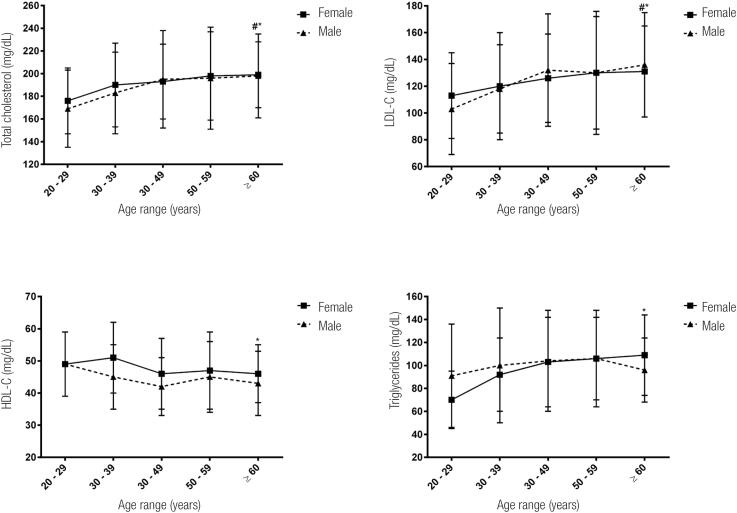
Test for significant linear trend between age range and total cholesterol, LDL-C, HDL-C and triglycerides. # men, *p* for trend < 0.05; *women, *p* for trend < 0.05.

A significant linear trend of increasing TC, LDL-C and TG levels as well as decreasing HDL-C levels with increasing age was observed among women (*p* for trend < 0.05) (Figure 1). On the other hand, a significant linear trend of increasing TC and LDL-C levels with increasing age was detected (*p* for trend < 0.01), but no significant linear trend was detected between age range and HDL-C and TG among men.

**Figure 2:**
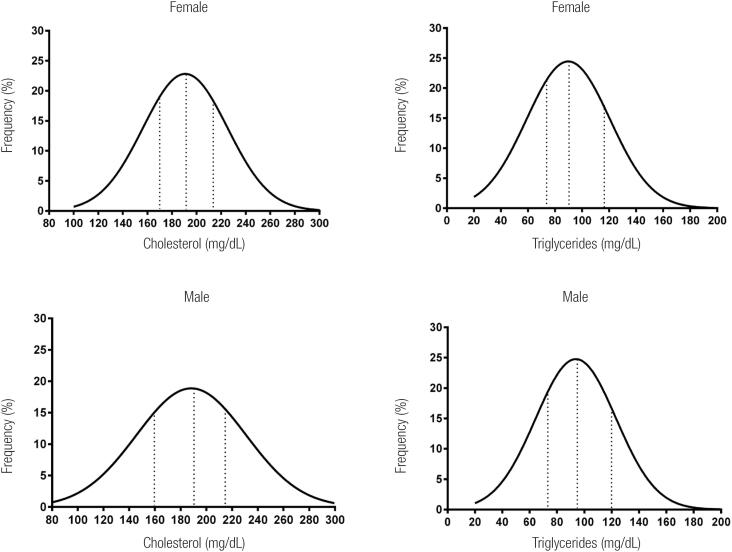
Nornal distribution curves of total cholesterol and triglycerides separately by gender. Dot lines indicate 25^th^, 50^th^ and 75^th^ percentiles from the left to the right.

Figure 2 shows the distribution curves of TC and TG for women and men. The medians with interquartile intervals for TC were 191 mg/dL (170215 mg/dL) and 189 mg/dL (159-217 mg/dL) in women and men, respectively. Likewise, the medians with corresponding interquartile intervals for TG were 91 mg/dL (75-118 mg/dL) and 96 mg/dL (74-120 mg/dL), respectively. The areas under the distribution curves provide the proportion of individuals with TC and TG above the 75^th^ percentile.

## DISCUSSION

The present study was carried out in Angolan civil servants, predominantly black and of middle/upper socioeconomic class. Higher frequencies of obesity, metabolic syndrome and low HDL-C were found in women than in men. On the other hand, obesity seems to cause the lipid profile to undergo greater changes in men than in women. Moreover, a significant linear trend of increasing TC and LDL-C levels as age increased was detected for both genders.

Epidemiologic studies have shown that elevated serum levels of cholesterol in the population increases the risk for coronary heart disease (21,22). Moreover, dyslipidemia, among other risk factors, has been implicated in the pathogenesis of stroke (23).

Data from the 2003 through 2006 NHANES survey were analyzed to determine the proportion of the US population with abnormalities in the lipid profile (24). Despite the existence of efficacious and cost-effective treatment guidelines, high prevalence of lipid abnormalities such as high LDL-C (27.0%), elevated TG levels (30.0%) and low HDL-C (23%) was reported. Although the study described here is not a population- based cohort, compared with the US population, Angolan civil servants have a lower proportion of high LDL-C (19.3%) and elevated TG (10.7%) and a much higher proportion of low HDL-C (49.8%), which is in great part affected by the huge proportion of low HDL-C observed in women (62.4%). Indeed, this finding is alarming given the strong association of low HDL-C with atherosclerosis (25).

An expert panel from the American College of Cardiology/American Heart Association recommended that all individuals over 20 years of age with LDL-C levels above 190 mg/dL should be encouraged to either start or continue statin use for prevention of coronary heart disease and stroke (26). However, statins are still widely underused in the clinical setting, and poor adherence to statin therapy has become a major concern for preventive cardiology (27).

Recently, a Finnish population-based study showed the cardiovascular burden of medication non-adherence (28). The rate of adherence to statins was 58% in men and 60% in women, which the authors classified as intermediate. In addition, compared with adherent patients, the adjusted odds ratio for stroke death in non-adherents was 2.04 (CI95% 1.72-2.43) at the year of death or at the end of follow-up.

Regarding African populations, most of the studies dealing with adherence to statin therapy were conducted among people of African descent living outside Africa. One of these studies reported that ethnicity (African American) was among the predictors of non-adherence to statin therapy (29). Moreover, among white and black Medicare beneficiaries discharged from the hospital following an ischemic stroke, the adherence to statin therapy was lower in African Americans than in white subjects (30).

Lack of access to medical care has been considered one of the plausible explanations for the differences in the adherence to statin therapy between black and white subjects. However, a previous study has shown that even without access barriers, African Americans were less adherent to medication therapies, including statins (31).

To our knowledge, the adherence rate to lipidlowering pharmacological therapy has not been documented in black African countries. Recently, a retrospective longitudinal study among Ghanaian heart failure patients has shown that statin users had a higher survival rate than those non-users who underwent another therapeutic option, but therapy adherence was not within the study's scope (32).

In the current study, around 60% of the volunteers claimed to have a good, or high, education level, and 68.1% comprised the middle or upper socioeconomic class. However, the mean values for the metabolic parameters were similar to those previously reported in the lower socioeconomic strata from black African populations (33). This result reflects the epidemiological transition of the Angolan population because even with enhanced socioeconomic status, the frequency of metabolic syndrome and elevated cholesterol levels remain meaningfully high, and this scenario tends to get worse as age advances.

Because this study was cross-sectional, it is not appropriate to establish a causal relationship between non-adherence to lipid-lowering therapy and cardiovascular risk factors. Indeed, this study was not designed to address this particular issue. In addition, as the sample was composed of public sector workers, the results should not be extrapolated to the whole Angolan population. However, it is worth wondering whether a high frequency of lipid disorders without treatment was observed in a population comprising almost two-thirds of the middle/upper-income segment and what to expect from a representative sample of the population comprising approximately 85% of the low-income class.

In summary, a high frequency of lipid disorders was observed among Angolan non-users of lipid-lowering medication. Longitudinal studies should be conducted to highlight the long-term consequences of non-treated lipid disorders in the Angolan population.
